# Prediction of pathological complete response after neoadjuvant therapy in breast cancer using pretreatment core biopsy pathology reports: a pathology-report-driven model with exploratory clinical-laboratory fusion

**DOI:** 10.3389/fonc.2026.1909071

**Published:** 2026-09-25

**Authors:** Rui Zhang, Luxia Qi, Bin Li, Guifang Zhang

**Affiliations:** 1 Department of Medical Oncology, Xinxiang Central Hospital, Xinxiang, Henan, China; 2 Department of Pain Medicine, Xinxiang Central Hospital, Xinxiang, Henan, China

**Keywords:** breast cancer, core biopsy, machine learning, multimodal prediction, natural language processing, neoadjuvant therapy, pathological complete response, pathology report

## Abstract

Pathological complete response (pCR) after neoadjuvant therapy is an important response endpoint in breast cancer. We investigated whether routinely generated pretreatment core biopsy pathology reports could support pCR prediction and whether clinical, preoperative laboratory, and treatment information provided incremental predictive value. This single-center retrospective model-development study included 171 patients with breast cancer who underwent core biopsy, neoadjuvant therapy, and surgery at Xinxiang Central Hospital between March 1, 2016, and January 1, 2026; 64 achieved pCR and 107 did not. We converted 361 pretreatment pathology files to patient-level text, extracted structured pathology phenotypes with a rule-based pipeline, and represented report text with character/marker n-gram term frequency-inverse document frequency followed by singular value decomposition. Five primary elastic net logistic regression models were assessed using 20 repeats of stratified 5-fold cross-validation, with all learned preprocessing confined to training folds. The clinical-laboratory model showed weak discrimination (area under the receiver operating characteristic curve [AUC] 0.420, 95% confidence interval [CI] 0.326-0.509). The structured pathology and report text models achieved AUCs of 0.738 (95% CI 0.664-0.810) and 0.689 (95% CI 0.606-0.767), respectively. Pathology fusion achieved an AUC of 0.747 (95% CI 0.672-0.815; Brier score 0.200, 95% CI 0.175-0.226). Adding clinical-laboratory variables did not improve AUC (multimodal AUC 0.733, 95% CI 0.656-0.805; difference vs. pathology fusion −0.014, 95% CI -0.048 to 0.017; P = 0.388). In a *post-hoc* secondary analysis, adding standardized regimen components to pathology fusion yielded an AUC of 0.757 (95% CI 0.679-0.824) and an AUC increase of 0.009 (95% CI −0.019 to 0.037; P = 0.487). The tested frozen BGE, Chinese RoBERTa, and ERNIE-Health embeddings did not outperform the lexical text representation. Among 20 audited image-only cases, OCR character accuracy was 98.72%; across 40 audited cases, critical pathology-field agreement ranged from 95.0% to 100%. Pretreatment pathology reports carried most of the observed predictive signal, whereas the incremental value of treatment information was small and imprecisely estimated. Independent external validation is required before clinical use.

## Introduction

Neoadjuvant systemic therapy is widely used for locally advanced and biologically high-risk early breast cancer (1–5). Pathological complete response (pCR) after neoadjuvant therapy is associated with improved long-term outcomes, particularly in biologically aggressive subtypes, and is widely used as a response endpoint in clinical trials and translational studies (1–5). Reliable pretreatment prediction of pCR could support research stratification, trial enrichment, and patient counseling, although a prediction model would require external and prospective validation before it could inform care.

Most pCR prediction studies have used imaging, digital pathology whole-slide images, manually curated immunohistochemistry (IHC), tumor microenvironment features, clinicopathologic models, or clinical-laboratory indices (6–21). Systematic reviews of MRI-based deep learning and multimodal prediction have highlighted rapid growth in this field, but most published models still depend on imaging infrastructures or curated multimodal registries that are not universally available (6, 15, 16). Quantitative digital histopathology from pretreatment core needle biopsy slides has shown strong predictive performance (10, 11), and image-analysis-based tumor-infiltrating lymphocyte quantification on pretreatment biopsy slides has also shown predictive value (12). Core biopsy biomarkers of the tumor microenvironment have been used to predict nodal pCR (13), and clinicopathologic or imaging nomograms have been developed in specific subgroups such as HER2-low disease or MRI-defined heterogeneous tumors (14–16). Peripheral blood-derived inflammatory and nutritional indices, including NLR, PLR, SII, SIRI, HALP, and related measures, have also been evaluated, but their predictive value has been inconsistent across cohorts and subtypes (17–21).

Routine pathology reports represent a different data source. They are widely available in real-world retrospective cohorts and contain both structured-like information (for example, ER, PR, HER2, and Ki-67) and narrative descriptors that may be difficult to capture in ordinary tabular datasets. Recent work has shown that rule-based natural language processing (NLP), conventional machine learning, and large language model pipelines can extract structured breast cancer biomarker data or clinical variables from narrative pathology reports at scale (22–27). Cheligeer and colleagues further demonstrated that large language models can detect pCR from population-based postoperative pathology reports (28). That task, however, is retrospective information extraction: the model reads reports written after surgery to determine whether pCR occurred. Pretreatment prediction is more difficult and clinically distinct because only information available before therapy should be used.

We therefore developed a pathology-report-driven framework for pCR prediction using pretreatment core biopsy reports. The framework combines two complementary report representations: rule-extracted structured pathology phenotypes and patient-level lexical text features from term frequency-inverse document frequency and singular value decomposition (TF-IDF/SVD). We further evaluated whether clinical and preoperative laboratory variables improved prediction when fused with report-derived features, analogous to multimodal prediction strategies explored in imaging-based studies (15, 16). Treatment information was excluded from the primary models to isolate the pathology-report signal. Because response is regimen-dependent, we also performed a *post-hoc* treatment-added secondary analysis and retained the primary estimates as regimen-marginal rather than regimen-specific predictions.

## Materials and methods

### Study design and cohort

This was a retrospective prediction modeling study using routinely collected clinical, laboratory, outcome, and pretreatment pathology report data from 171 hospitalized patients with breast cancer at Xinxiang Central Hospital who underwent pretreatment core biopsy, neoadjuvant therapy, and surgery between March 1, 2016, and January 1, 2026. The analytic cohort included 64 patients with pCR and 107 without pCR.

### Eligibility criteria

Patients were eligible if they met all of the following criteria: (1) pathologically confirmed breast cancer; (2) hospitalization at Xinxiang Central Hospital with a documented treatment sequence of pretreatment core biopsy, neoadjuvant therapy, and subsequent surgery between March 1, 2016, and January 1, 2026; (3) availability of at least one pretreatment core biopsy pathology report that could be linked to the study patient; (4) availability of postoperative response information sufficient for pCR adjudication; and (5) availability of linked clinical and preoperative laboratory data.

Patients were not included in the analytic cohort if they lacked a matched pretreatment core biopsy pathology report, if available pathology files could not be confirmed as pretreatment biopsy material, if the temporal sequence of biopsy, neoadjuvant therapy, and surgery could not be verified from the record, if postoperative response status remained unavailable or ambiguous after chart re-review, if identifiers were insufficient to link clinical and pathology records, or if duplicate records were present. The final analytic cohort comprised 171 patients who met all eligibility criteria.

Treatment regimen variables were not included in the primary analysis dataset by design. This preserved the main emphasis on pretreatment pathology and reduced the possibility that the primary model would learn institution-specific treatment allocation. It does not eliminate confounding by regimen, because pCR is intrinsically treatment-dependent, and the primary estimates should therefore be interpreted as regimen-marginal associations within the treatments used at this institution rather than regimen-specific response probabilities.

For a *post-hoc* treatment-added secondary analysis, a patient-linked regimen field was available for all 171 patients. The 81 distinct free-text entries were standardized without reference to pCR outcome into anthracycline exposure, platinum exposure, anti-HER2 category (none documented, trastuzumab plus pertuzumab, or trastuzumab plus pyrotinib), immunotherapy exposure, and endocrine/CDK4/6 exposure. A locked coding dictionary normalized capitalization, punctuation, regimen abbreviations, and explicit drug names. Eight patient records contained abbreviations that did not permit complete component resolution; these were coded conservatively using only recognizable regimen abbreviations or explicit drug names. Dose, dose intensity, treatment delay, completion, and cycle count were not modeled because these details were not recorded uniformly and the source field mixed concise regimen labels with narrative administration descriptions.

Because this was a retrospective study, postoperative response had already been assessed during routine care by the treating physician and department director. For the study dataset, the study initiator re-reviewed the patient record and surgical pathology documents to confirm the response label. Cases with unresolved ambiguity after re-review were excluded. The final analytic outcome was the curated consensus postoperative response field (pCR vs. non-pCR). The postoperative ypTN field was used only for audit and not as a model input. For modeling, pCR was operationalized as absence of residual invasive disease in the breast and axillary nodes, corresponding to ypT0/is ypN0-equivalent notations when stage data were available.

### Pathology report conversion

Pretreatment core biopsy pathology files were stored as PDFs and image files. A total of 361 source files were mapped to the 171 included patients, comprising 234 PDFs and 127 images. PDF files were processed by text-layer extraction when a usable text layer was present. Image files were converted to text by optical character recognition (OCR). Conversion quality checks assessed whether a non-empty clinically interpretable text output was produced and recorded the OCR engine’s mean confidence as a processing diagnostic. All text fragments belonging to the same patient were concatenated in source order into a patient-level report document. Patient-level pathology text coverage was complete in the analytic cohort (171/171). The median report text length was 723 characters (interquartile range 563-959). A supplementary archive search during revision retrieved 15 additional pretreatment IHC reports for 14 patients already in the analytic cohort; one duplicate primary diagnostic report was excluded. No patients or outcomes were added or changed, and all analyses were rerun using the updated report corpus.

Report conversion outputs were retained internally for quality control. Public supplementary materials include only deidentified aggregate conversion quality-control summaries; source-file paths, patient-level report text, and raw report excerpts are not included. Postoperative pathology text was not used as a predictor.

### Structured pathology phenotype extraction

Rule-based extraction was applied to patient-level pretreatment report text. The rules were developed iteratively from recurring syntax in deidentified institutional report formats without access to pCR labels. Development began with clinically defined marker names and common aliases, followed by normalization of full-width punctuation, plus/minus signs, whitespace, and letter case. Marker-specific regular expressions then parsed parenthetical expressions for ER, PR, HER2, Ki-67, and AR; numeric values were constrained to 0%-100%, and textual positive, negative, or uncertain qualifiers were resolved with explicit precedence rules. Dictionary searches captured CK5/6, EGFR, p53, E-cadherin, GATA3, invasive carcinoma, non-special type, ductal carcinoma *in situ*, lobular terms, lymph-node or axillary wording, node-positive wording, multifocality, HER2 heterogeneity, FISH recommendation, and SBR grade. During development, recurrent formatting failures were identified by inspection and the normalization and marker-alias rules were refined; the final rule dictionary was then locked before the revised model analyses. No pathologist formally participated in rule development, and no formal expert-annotated development set was used to refine the rules. The blinded clinical audit described below was conducted only after the rules were locked and was not used to refine the rule set. No outcome-based rule or predictor selection was performed.

When multiple pretreatment fragments were available for a patient, the patient-level summary retained the highest reported ER, PR, and Ki-67 percentages and the highest HER2 IHC category. The rule-based subtype included HER2-positive/HR-positive, HER2-positive/HR-negative, Luminal B-like, Luminal A or low-proliferation-like, TNBC-like, and insufficient marker data. A deidentified rule and variable-coding dictionary is provided in Supplementary File S1. Adaptation at another institution would require local terminology and template mapping, testing against a blinded locally annotated sample, correction of systematic discrepancies, and relocking of the dictionary before external evaluation; portability should not be assumed from the present single-center corpus.

For ER, PR, and AR, an explicitly negative status with no percentage was treated as a structural omission and coded as 0%; it was not treated as an unknown value. The single report containing “Ki-67(-)” was likewise coded as 0%. Truly unreported or uncertain marker percentages remained missing. A combined indicator identified incomplete key IHC information (ER, PR, or Ki-67), with separate indicators for unknown AR percentage and HER2 category. These indicators were included to retain potentially informative report-completeness patterns, although such patterns may also encode institution-specific documentation practices.

To assess downstream extraction fidelity, 40 patients were sampled with 10 cases per cell defined by source modality (image-only vs. PDF-only) and outcome (pCR vs. non-pCR). Report folders were relabeled with audit identifiers, and a medical oncologist with more than 10 years of clinical experience reviewed the source reports while blinded to automated values, model predictions, and outcomes. ER, PR, HER2, Ki-67, and AR results were abstracted using the prespecified patient-level aggregation rules after the extraction pipeline had been locked. Exact agreement and Cohen’s kappa were calculated for categorical fields; availability agreement, exact agreement, agreement within 10 percentage points, and mean absolute error were calculated for numeric fields. Numeric agreement concerned reported percentages before the subsequent structural-negative recoding used for modeling. For the 20 image-only cases, the reviewer also transcribed the pathology diagnosis or supplement and IHC text from reports containing ER, PR, HER2, Ki-67, or AR; reports without IHC results were outside the defined character-audit scope. The matched manual and OCR text scope was confirmed while the reviewer remained blinded to OCR output and before CER was calculated. Manual and OCR strings underwent Unicode NFKC normalization, lowercasing, whitespace removal, and canonicalization of dash variants, while clinically relevant symbols including +, −, and % were retained. Character error rate (CER) was defined as Levenshtein edit distance divided by the number of manually transcribed reference characters. A 95% CI for the micro-averaged CER was obtained by 20,000 case-level bootstrap resamples.

### Clinical and laboratory variables

The clinical-laboratory feature set included age, BMI, tumor location, clinical grade/stage fields, and laboratory variables available before surgery. Derived inflammatory indices included neutrophil-to-lymphocyte ratio (NLR), platelet-to-lymphocyte ratio (PLR), lymphocyte-to-monocyte ratio (LMR), and systemic immune-inflammation index (SII). Other laboratory variables included CRP, LDH, albumin, glucose, BUN, creatinine, platelets, and hemoglobin when available. The laboratory block therefore represents preoperative testing rather than strictly pretreatment biology, and its use in the multimodal model was exploratory. Clinical and laboratory fields were nearly complete, with tumor location missing in 3/171 patients. After structural negative results were coded as 0%, ER, PR, and Ki-67 percentages were available for all 171 patients; AR percentage remained truly unknown in 33/171 patients. HER2 IHC remained uncertain in 3/171 reports because the source text contained an unresolved 0/O character ambiguity.

### Text feature construction

The primary report text representation used a custom tokenizer designed for Chinese pathology reports. The tokenizer retained Chinese characters, Latin marker tokens, percentage tokens, and HER2 plus-sign patterns. TF-IDF features were generated using 1- to 3-grams, with a minimum document frequency of 2 and a maximum of 3,000 features. TruncatedSVD reduced the TF-IDF matrix to 25 components, followed by standardization. The TF-IDF and SVD steps were fitted only within training folds and applied to corresponding held-out folds.

Three frozen pretrained encoders were evaluated as sensitivity analyses: the general-purpose Chinese BAAI/bge-small-zh-v1.5 embedding model (29), Chinese RoBERTa with whole-word masking (30), and the Chinese biomedical ERNIE-Health encoder (31). Reports were tokenized without translation and split at each model’s 512-token limit. For RoBERTa and ERNIE-Health, hidden states were mean-pooled over non-special tokens; chunk embeddings were L2-normalized, averaged per patient, and normalized again. BGE used its corresponding frozen chunk-embedding procedure. For every encoder, standardization and principal component analysis to 25 dimensions were fitted only in each training fold. English BioBERT and ClinicalBERT were not used because the reports were written in Chinese and translation would have introduced an additional unvalidated preprocessing step. Chinese MedBERT was not evaluated in this revision; the transformer comparison is therefore a limited, language-matched exploratory benchmark rather than an exhaustive comparison of biomedical language models.

### Model development

Five feature-set models were evaluated:

1. Model A: clinical and laboratory variables.
2. Model B: structured pathology phenotypes.
3. Model C: report text TF-IDF/SVD features.
4. Model D: structured pathology phenotypes plus report text features.
5. Model E: clinical-laboratory variables plus structured pathology phenotypes plus report text features.

Two *post-hoc* secondary models were then evaluated: Model F added the five standardized treatment variables to Model D, and Model G added them to Model E. Treatment variables were processed within each training fold. On full-cohort transformation solely for dimensionality reporting, Models F and G contained 93 and 135 features, respectively.

All models used elastic net logistic regression with the SAGA solver, using scikit-learn’s l1_ratio as the Elastic Net mixing parameter (alpha), C = 0.2 as the inverse regularization-strength parameter, a maximum of 10,000 iterations, and balanced class weights. The implementation parameterized penalty strength with C rather than a separately optimized lambda; the primary analysis used the common fixed setting l1_ratio = 0.5 and C = 0.2 for model comparability, without selecting either parameter using held-out outcomes. These values were fixed for the primary analysis and were not selected using validation-fold outcomes. Numeric medians, scaling parameters, categorical modes, one-hot levels, TF-IDF vocabulary and weights, SVD loadings, and embedding PCA loadings were learned only from each training fold and applied to its held-out fold. No univariable outcome-based feature screening was used. After transformation on the full cohort solely for dimensionality reporting, Models A-E contained 42, 61, 25, 86, and 128 features, respectively; fold-specific one-hot dimensions could vary slightly.

### Validation and statistical analysis

Model performance was evaluated using 20 repeats of stratified 5-fold cross-validation. For each patient, out-of-fold probabilities from repeated test-fold assignments were averaged to form a patient-level predicted probability. Discrimination was assessed using AUC and average precision. Classification metrics at a probability threshold of 0.5 included accuracy, sensitivity, specificity, precision, and F1 score. Calibration was assessed using the Brier score and calibration curves. Decision curve analysis was generated for exploratory clinical utility assessment.

Bootstrap resampling with 2,000 replicates estimated 95% confidence intervals for AUC, average precision, F1, and Brier score. Paired bootstrap tests compared AUC between prespecified model pairs: structured pathology vs. clinical-laboratory, report text vs. clinical-laboratory, pathology fusion vs. structured pathology, pathology fusion vs. report text, multimodal fusion vs. pathology fusion, and multimodal fusion vs. clinical-laboratory. P values below 0.05 were considered statistically significant. These pairwise comparisons were exploratory because all models were evaluated within the same internally resampled cohort. No external validation cohort was available.

All eligible patients were included, and no *a priori* sample-size calculation was performed for this retrospective study. The cohort contained 171 patients and 64 pCR events. Contemporary guidance emphasizes that prediction-model sample size should account for outcome frequency, anticipated model fit, and overfitting rather than rely on a simple events-per-variable threshold (32). The present event count remained limited relative to the transformed feature spaces, particularly for the fusion models; penalization, low-dimensional text projections, fold-contained preprocessing, and repeated cross-validation were used to constrain model complexity. We did not calculate observed *post-hoc* power because it is a transformation of the observed effect and adds little beyond the estimate and its confidence interval (33). The widths of the bootstrap confidence intervals and paired AUC-difference intervals were therefore used to communicate precision.

Four sensitivity analyses assessed modeling choices. First, BGE, Chinese RoBERTa, and ERNIE-Health embeddings replaced TF-IDF/SVD under the same 20-repeat 5-fold framework. Second, the three patients with uncertain HER2 IHC were excluded, leaving a HER2-resolved sensitivity subset of 168 patients; AR remained unknown in 33 patients and was not treated as resolved by this analysis. Third, class weights were removed. Fourth, all five principal models underwent nested tuning with five repeats of 5-fold outer cross-validation and 3-fold inner cross-validation; the inner grid included l1_ratio values 0, 0.5, and 1.0 and C values 0.05, 0.2, 1.0, and 5.0. Models F and G underwent the same repeated cross-validation, unweighted analysis, and nested tuning. *Post-hoc* paired bootstrap comparisons evaluated Model F vs. Model D and Model G vs. Model E. All imputation and feature transformations remained inside the corresponding training or inner-tuning folds.

For structured-pathology interpretability, standardized elastic net coefficients were aggregated across the 100 training folds of the main repeated cross-validation. We report selection frequency, median coefficient, interquartile range, mean absolute coefficient, and mean absolute contribution to held-out log odds. Because correlated one-hot variables can share or exchange coefficients, these summaries describe model behavior rather than causal biomarker effects. SVD and PCA components were not assigned semantic labels.

Reporting followed the intent of TRIPOD+AI for clinical prediction model studies (34). A completed working checklist is provided as supplementary submission material.

## Results

### Cohort and pathology report corpus

The analytic cohort included 171 patients, of whom 64 (37.4%) had pCR and 107 (62.6%) did not. Pretreatment pathology report conversion produced complete patient-level text coverage. The 361 source pathology files included 234 PDFs and 127 images, with one to five report fragments per patient. All 234 PDFs yielded usable text layers, and all 127 images passed the prespecified OCR text-output quality checks. For images, the mean, median, and minimum OCR engine confidence values were 0.9768, 0.9773, and 0.9603, respectively. These confidence values are engine diagnostics and should not be interpreted as character-level accuracy.

Clinical and laboratory predictors retained for Model A were nearly complete, with missingness limited to tumor location in 3/171 patients. ER, PR, and Ki-67 percentages were available for all 171 patients after archived IHC retrieval and structural-negative recoding. AR percentage remained unknown for 33 patients, and HER2 IHC remained uncertain for three patients because HER2(O) could not be distinguished confidently from HER2(0) in the source text. Ambiguous values were not manually converted to 0.

Treatment regimen information was present for all 171 patients. Documented regimens included anthracycline exposure in 74 patients and platinum exposure in 77. Anti-HER2 treatment was coded as trastuzumab plus pertuzumab in 83 patients, trastuzumab plus pyrotinib in 7, and none documented in 81. Immunotherapy and endocrine/CDK4/6 components were documented in two and six patients, respectively. The latter strata were too small for reliable treatment-specific subgroup estimates.

In the 20 image-only audit cases, 39 edits were observed across 3,047 manually transcribed reference characters, corresponding to a micro-averaged CER of 1.28% (case-bootstrap 95% CI 0.87%-1.69%) and character accuracy of 98.72%. The median case-level CER was 1.08% (IQR 0%-1.89%; maximum 3.37%). Across all 40 audit cases, the exact agreement was 100% for ER status, PR status, and HER2 IHC category (Cohen’s kappa = 1.000 for each) and 95.0% for AR status (38/40; 95% CI 83.5%-98.6%; kappa = 0.913). AR status agreement was 90.0% in image-only cases and 100% in PDF-only cases. Numeric-value availability agreement was 100% for each marker. Among available paired values, the exact agreement was 100% for ER (18/18), PR (13/13), Ki-67 (40/40), and AR (21/21) percentages; all paired differences were within 10 percentage points, and the mean absolute error was 0 percentage points. Detailed deidentified results are provided in **Supplementary Table S5** and **Supplementary File S4**.

Baseline age was similar between groups (median 51.5 years in pCR vs. 52.0 years in non-pCR; P = 0.354). BMI, NLR, PLR, LMR, SII, CRP, LDH, albumin, glucose, hemoglobin, and platelet count did not differ significantly by pCR status in descriptive univariable comparisons. In contrast, pathology-related variables differed between groups. Median ER was 0% in the pCR group and 80% in the non-pCR group (P < 0.001), median PR was 0% and 20%, respectively (P < 0.001), and median Ki-67 was 40% and 30%, respectively (P = 0.045). HER2 IHC category was associated with pCR (P < 0.001), with HER2 IHC 3+ present in 42/64 pCR cases and 34/107 non-pCR cases. The rule-based subtype was also associated with pCR (P < 0.001). These comparisons were descriptive and were not used for feature selection.

Key cohort characteristics are shown in **Table 1**. The full baseline table is available as **Supplementary Table S1**.

**Table 1:** Key baseline and pathology characteristics by pCR status.

Characteristic	Overall (n=171)	pCR (n=64)	non-pCR (n=107)	P value
Age, years, median (IQR)	52.0 (45.0-60.0)	51.5 (44.5-57.8)	52.0 (45.0-61.0)	0.354
BMI, kg/m^2^, median (IQR)	25.5 (23.5-27.6)	25.6 (23.0-27.6)	25.5 (23.6-27.6)	0.688
NLR, median (IQR)	3.0 (2.2-4.0)	3.2 (2.3-3.8)	3.0 (2.2-4.2)	0.773
PLR, median (IQR)	151.6 (115.4-194.4)	147.7 (116.9-187.3)	152.9 (114.9-194.4)	0.922
LMR, median (IQR)	2.7 (2.0-3.2)	2.5 (1.9-3.1)	2.7 (2.0-3.2)	0.386
SII, median (IQR)	738.5 (518.5-1106.4)	706.4 (490.0-1048.0)	752.8 (522.7-1120.4)	0.240
CRP, median (IQR)	15.2 (12.0-19.6)	14.5 (10.9-17.1)	16.5 (12.4-21.2)	0.160
LDH, median (IQR)	222.8 (163.2-288.8)	206.0 (156.4-264.6)	235.7 (167.6-304.7)	0.203
Albumin, median (IQR)	41.7 (38.3-44.7)	41.2 (38.8-44.3)	41.7 (38.3-45.1)	0.953
Hemoglobin, median (IQR)	14.4 (13.2-15.1)	14.3 (13.1-15.1)	14.4 (13.2-15.1)	0.592
Platelets, median (IQR)	243.0 (209.0-293.0)	228.5 (188.8-285.0)	248.0 (213.0-293.0)	0.105
ER, %, median (IQR)	55.0 (0.0-90.0)	0.0 (0.0-70.0)	80.0 (0.0-90.0)	<0.001
PR, %, median (IQR)	3.0 (0.0-60.0)	0.0 (0.0-10.0)	20.0 (0.0-80.0)	<0.001
Ki-67, %, median (IQR)	40.0 (25.0-60.0)	40.0 (30.0-60.0)	30.0 (20.0-50.0)	0.045
HER2 IHC 3+, n (%)	76 (44.4%)	42 (65.6%)	34 (31.8%)	<0.001
HER2 IHC 2+, n (%)	39 (22.8%)	5 (7.8%)	34 (31.8%)	
HER2 IHC 0/1+, n (%)	53 (31.0%)	16 (25.0%)	37 (34.6%)	
HER2 IHC missing/uncertain, n (%)	3 (1.8%)	1 (1.6%)	2 (1.9%)	
Rule-based HER2-positive/HR-positive subtype, n (%)	47 (27.5%)	26 (40.6%)	21 (19.6%)	<0.001
Rule-based luminal B-like subtype, n (%)	56 (32.7%)	11 (17.2%)	45 (42.1%)	
Rule-based HER2-positive/HR-negative subtype, n (%)	29 (17.0%)	16 (25.0%)	13 (12.1%)	

Explicitly negative ER or PR status without a numeric percentage was coded as 0%. ER, PR, and Ki-67 were available for all patients after archived-report retrieval; the three uncertain HER2 values were retained as uncertain. The full baseline table, including all categorical levels and additional laboratory variables, is provided as **Supplementary Table S1**. P values are descriptive and are from Mann–Whitney U tests for continuous variables and chi-square/Fisher exact tests for categorical variables; they were not used for feature selection.

### Main model performance

Model A, using clinical-laboratory variables alone, showed weak discrimination with an AUC of 0.420 (95% CI 0.326 to 0.509), average precision of 0.326 (95% CI 0.255 to 0.425), F1 score of 0.360 (95% CI 0.259 to 0.455), and Brier score of 0.266 (95% CI 0.254 to 0.280).

Pathology-derived models performed better. Model B, using rule-extracted structured pathology phenotypes, achieved an AUC of 0.738 (95% CI 0.664-0.810). Model C, using report text TF-IDF/SVD features, achieved an AUC of 0.689 (95% CI 0.606-0.767). Compared with Model A, the AUC difference was 0.318 for structured pathology (95% CI 0.206-0.432; paired bootstrap P < 0.001) and 0.270 for report text (95% CI 0.147-0.391; P < 0.001).

Model D, combining structured pathology phenotypes with report text features, achieved an AUC of 0.747 (95% CI 0.672-0.815). Its average precision was 0.591 (95% CI 0.473-0.717), the F1 score was 0.620 (95% CI 0.526-0.707), and the Brier score was 0.200 (95% CI 0.175-0.226). The AUC difference for Model D versus Model B was 0.009 (95% CI −0.035 to 0.053; P = 0.725), and that versus Model C was 0.058 (95% CI 0.002 to 0.112; P = 0.040).

Model E, adding clinical-laboratory variables to the pathology fusion model, achieved an AUC of 0.733 (95% CI 0.656-0.805), average precision of 0.583 (95% CI 0.466-0.709), F1 score of 0.582 (95% CI 0.478-0.671), and Brier score of 0.205 (95% CI 0.178-0.233). Compared with Model D, Model E did not improve AUC (difference −0.014, 95% CI −0.048 to 0.017; P = 0.388). Model performance is summarized in **Table 2** and **Figure 1**, and the prespecified paired bootstrap AUC comparisons are shown in **Table 3**.

**Table 2:** Main repeated cross-validation model performance.

Model	AUC (95% CI)	Average precision (95% CI)	Accuracy	Sensitivity	Specificity	Precision	F1 (95% CI)	Brier score (95% CI)
Model A: clinical/laboratory	0.420 (0.326-0.509)	0.326 (0.255-0.425)	0.439	0.422	0.449	0.314	0.360 (0.259-0.455)	0.266 (0.254-0.280)
Model B: structured pathology	0.738 (0.664-0.810)	0.577 (0.458-0.714)	0.667	0.734	0.626	0.540	0.623 (0.523-0.706)	0.206 (0.183-0.230)
Model C: report text TF-IDF/SVD	0.689 (0.606-0.767)	0.544 (0.436-0.673)	0.626	0.625	0.626	0.500	0.556 (0.455-0.646)	0.220 (0.200-0.239)
Model D: pathology structured + text	0.747 (0.672-0.815)	0.591 (0.473-0.717)	0.684	0.688	0.682	0.564	0.620 (0.526-0.707)	0.200 (0.175-0.226)
Model E: multimodal	0.733 (0.656-0.805)	0.583 (0.466-0.709)	0.655	0.641	0.664	0.532	0.582 (0.478-0.671)	0.205 (0.178-0.233)

**Figure 1:**
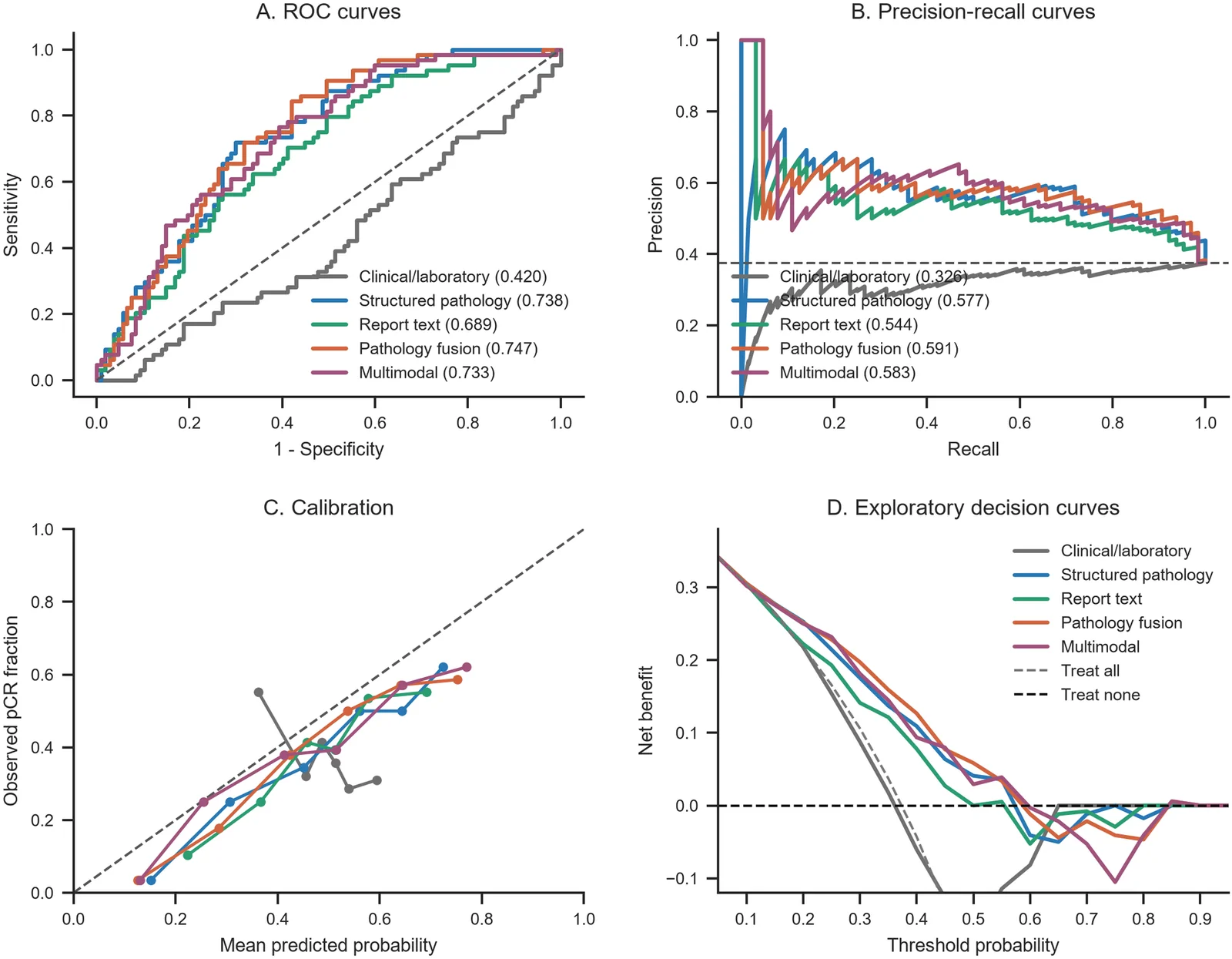
Main model performance. ROC curves, precision -recall curves, calibration curves, and decision curve analysis for the five primary models: clinical-laboratory, structured pathology, report text TF-IDF/SVD, pathology structured plus text, and multimodal fusion.

**Table 3:** Prespecified paired bootstrap AUC comparisons.

Comparison	AUC difference	95% CI	Bootstrap P value
Structured pathology vs. clinical/laboratory	0.318	0.206 to 0.432	<0.001
Report text TF-IDF/SVD vs. clinical/laboratory	0.270	0.147 to 0.391	<0.001
Pathology structured + text vs. structured pathology	0.009	-0.035 to 0.053	0.725
Pathology structured + text vs. report text TF-IDF/SVD	0.058	0.002 to 0.112	0.040
Multimodal vs. pathology structured + text	-0.014	-0.048 to 0.017	0.388
Multimodal vs. clinical/laboratory	0.313	0.213 to 0.410	<0.001

### Sensitivity analyses

In frozen-encoder sensitivity analyses, text-only BGE, Chinese RoBERTa, and ERNIE-Health models achieved AUCs of 0.546 (95% CI 0.454-0.639), 0.639 (95% CI 0.555-0.724), and 0.620 (95% CI 0.532-0.705), respectively. The corresponding structured-pathology fusion AUCs were 0.694 (95% CI 0.614-0.769), 0.738 (95% CI 0.661-0.808), and 0.720 (95% CI 0.644-0.795). None exceeded the TF-IDF/SVD text model (AUC 0.689) or its pathology fusion (AUC 0.747). Compared with TF-IDF/SVD, the text-only AUC differences were −0.144 for BGE (95% CI −0.224 to −0.062; P = 0.001), −0.050 for Chinese RoBERTa (95% CI −0.113 to 0.012; P = 0.117), and −0.069 for ERNIE-Health (95% CI −0.152 to 0.008; P = 0.075).

In the HER2-resolved sensitivity subset, 168 patients remained (63 with pCR and 105 without pCR). The clinical-laboratory, structured pathology, report text, pathology fusion, and multimodal models achieved AUCs of 0.462 (95% CI 0.379-0.555), 0.745 (95% CI 0.673-0.820), 0.689 (95% CI 0.604-0.769), 0.756 (95% CI 0.680-0.825), and 0.742 (95% CI 0.664-0.815), respectively.

Removing balanced class weights produced similar AUCs, including 0.746 for pathology fusion and 0.734 for multimodal fusion. In nested tuning of all five principal models, AUCs were 0.404 for clinical-laboratory, 0.727 for structured pathology, 0.683 for report text, 0.745 for pathology fusion, and 0.717 for multimodal fusion. The similarity of the fixed-parameter and nested-tuning estimates did not suggest that the main findings were driven by the prespecified Elastic Net settings, although the nested analysis used fewer outer repeats and remained internal.

Across 100 training folds, PR percentage and ER percentage were selected in every structured-pathology model and had negative median standardized coefficients (−0.469 and −0.359). HER2 IHC 3+ was also selected in every fold with a positive median coefficient (0.520), whereas Ki-67 percentage was selected in 97% of folds with a positive median coefficient (0.153). The HER2-positive/HR-positive subtype was selected in every fold with a positive median coefficient (0.485). Because key IHC status was complete after the archive update, the key-IHC-missingness indicator was constant and had no contribution. Full coefficient stability results are shown in **Supplementary Table S7** and **Figure 2**. .

**Figure 2:**
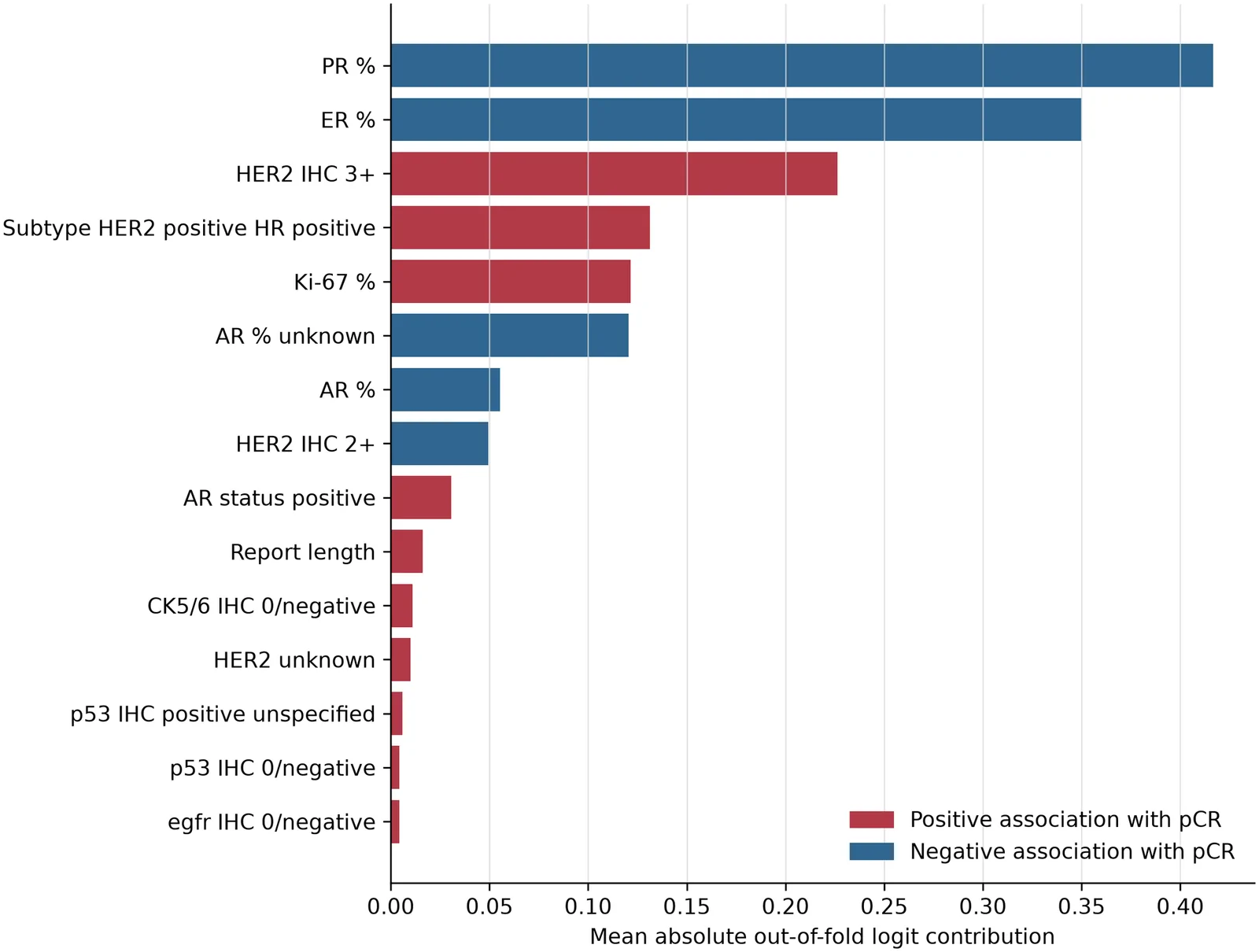
Fold-aggregated structured-pathology feature contributions. Selection frequency and median standardized Elastic Net coefficient with interquartile range across 100 training folds. Positive coefficients increase predicted pCR log odds; negative coefficients decrease predicted pCR log odds. Coefficients describe model behavior and are not causal effects.

### Treatment-added secondary analysis

Adding treatment variables to pathology fusion increased AUC from 0.747 to 0.757 (95% CI 0.679-0.824), with average precision increasing from 0.591 to 0.624 and Brier score decreasing from 0.200 to 0.197. The paired AUC increase was 0.009 (95% CI −0.019 to 0.037; P = 0.487). Adding treatment to the clinical-laboratory-pathology multimodal model increased AUC from 0.733 to 0.739 (95% CI 0.664-0.809); the paired increase was 0.006 (95% CI −0.019 to 0.033; P = 0.690). Thus, treatment information produced a small numerical gain, but the confidence intervals were compatible with no gain.

The direction was similar without class weights: pathology fusion AUC increased from 0.746 to 0.756 after treatment was added. In nested tuning, pathology fusion plus treatment achieved an AUC of 0.752 compared with 0.745 for pathology fusion alone (difference 0.007, 95% CI −0.023 to 0.038; P = 0.693). Corresponding results are shown in **Table 4** and **Supplementary Table S9**.

**Table 4:** Treatment-added secondary analysis.

Analysis	Reference model AUC (95% CI)	Treatment-added model AUC (95% CI)	Paired AUC difference (95% CI)	Bootstrap P value
Main: pathology fusion vs. pathology fusion + treatment	0.747 (0.672-0.815)	0.757 (0.679-0.824)	0.009 (-0.019 to 0.037)	0.487
Main: multimodal vs. multimodal + treatment	0.733 (0.656-0.805)	0.739 (0.664-0.809)	0.006 (-0.019 to 0.033)	0.690
Unweighted: pathology fusion vs. pathology fusion + treatment	0.746 (0.671-0.815)	0.756 (0.679-0.823)	0.010 (-0.020 to 0.039)	0.512
Nested tuning: pathology fusion vs. pathology fusion + treatment	0.745 (0.668-0.814)	0.752 (0.677-0.822)	0.007 (-0.023 to 0.038)	0.693

Treatment-added models were *post-hoc* secondary analyses. Their predictions are associational and should not be interpreted as treatment-effect estimates.

### Visual summary

**Figure 3** presents the study workflow. **Figure 1** shows ROC, precision-recall, calibration, and exploratory decision curves for the five principal models. **Figure 4** summarizes the outcome distribution and pathology-related signal patterns. **Figures 5**, **6** show the main AUC estimates and paired AUC differences. **Figure 2** presents fold-aggregated structured-feature coefficients, and **Figure 7** compares the lexical and frozen-encoder NLP baselines.

**Figure 3:**
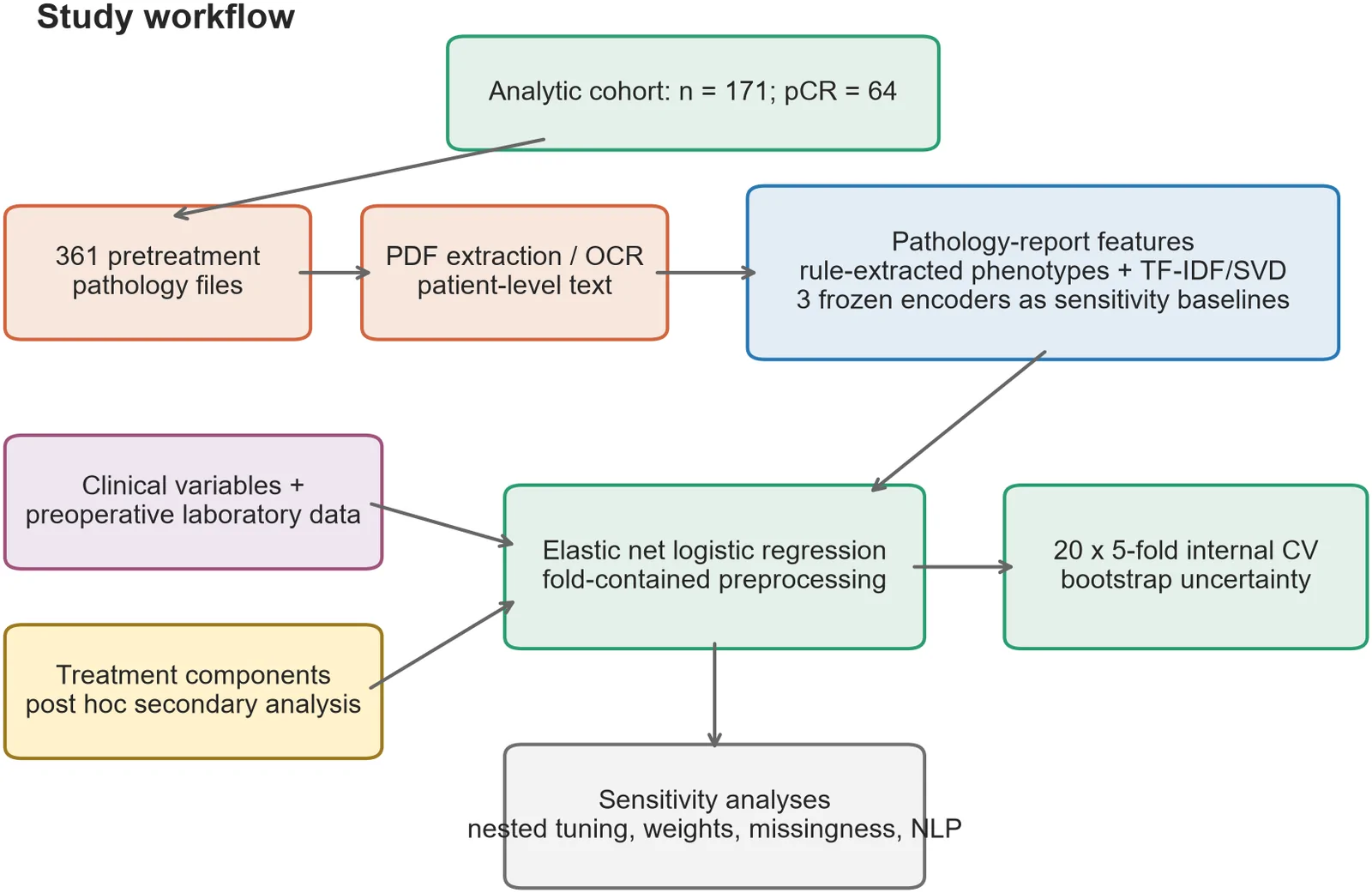
Study workflow and feature construction. The workflow shows the 171-patient analytic cohort, conversion of PDF and image reports to patient-level text, pathology-report representations, clinical-laboratory feature processing, *post-hoc* treatment-added secondary analysis, and repeated cross-validation model evaluation.

**Figure 4:**
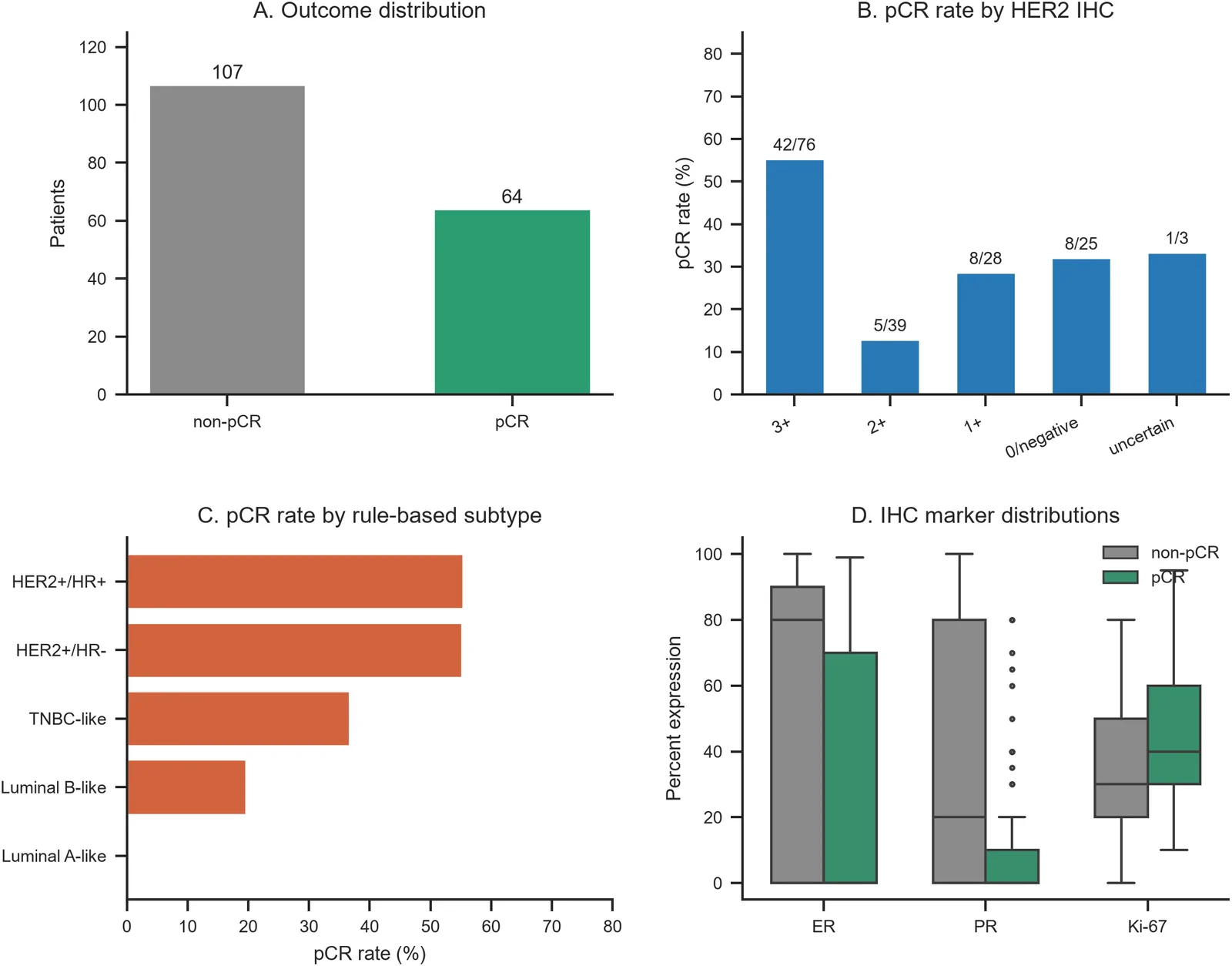
Pathology-related signal patterns. Summary of pCR distribution and pathology-derived characteristics, including receptor-related and subtype-related differences by outcome.

**Figure 5:**
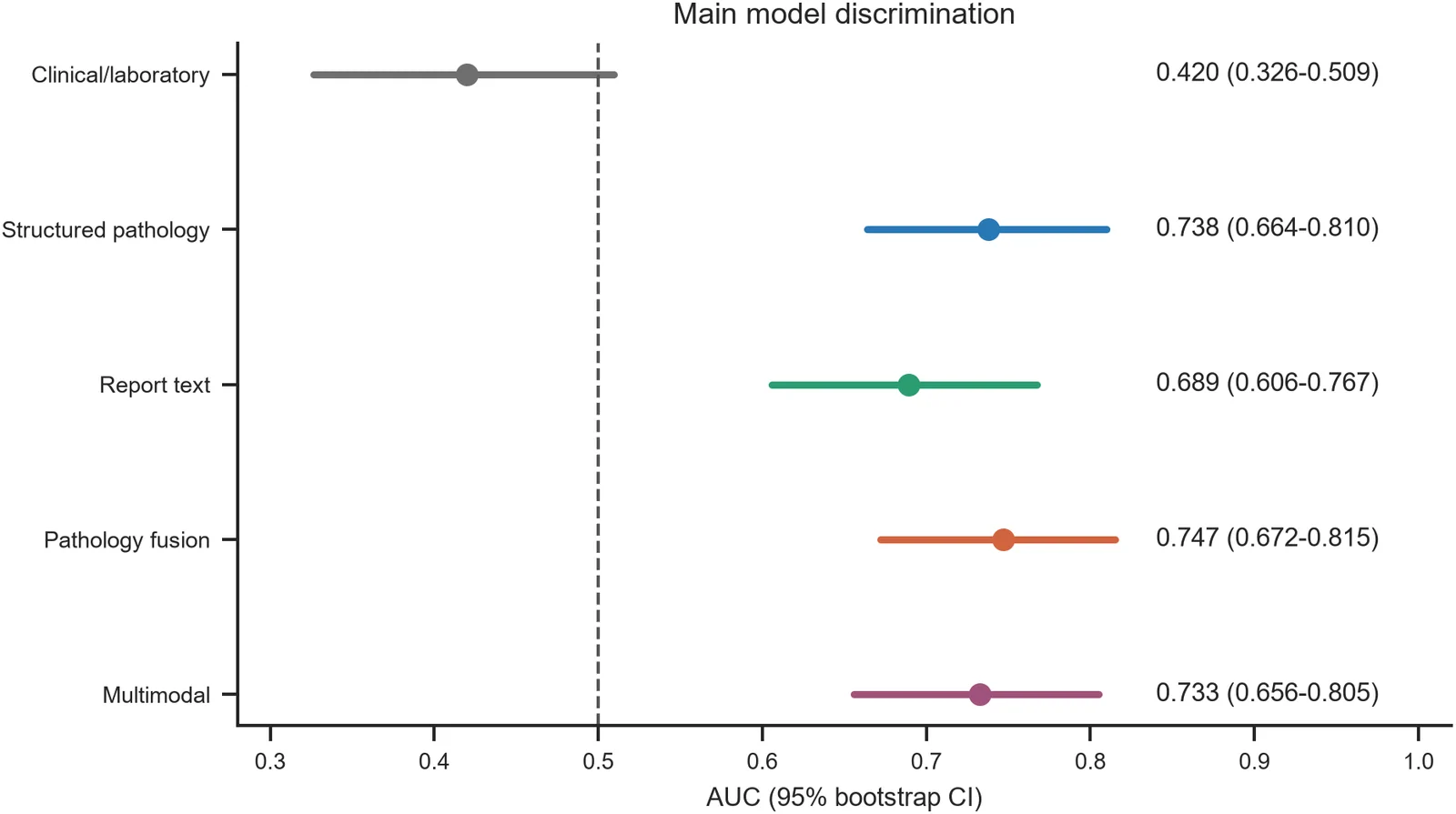
Main-analysis AUC forest plot. AUC estimates with bootstrap 95% confidence intervals for the five primary models. The dashed vertical line indicates AUC = 0.5.

**Figure 6:**
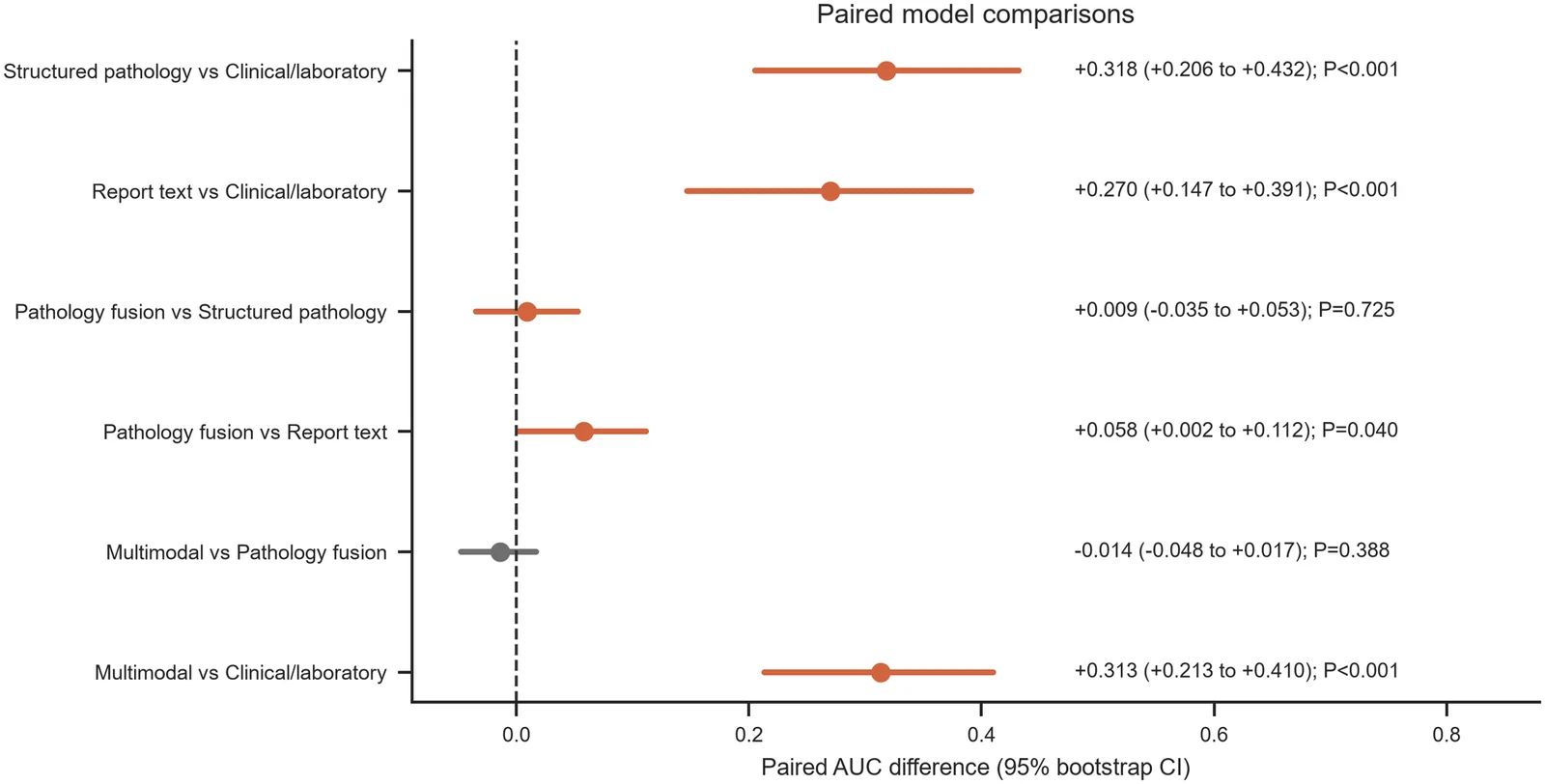
Paired bootstrap AUC comparisons. Prespecified paired AUC differences with bootstrap 95% confidence intervals and P values. The vertical line indicates no AUC difference.

**Figure 7:**
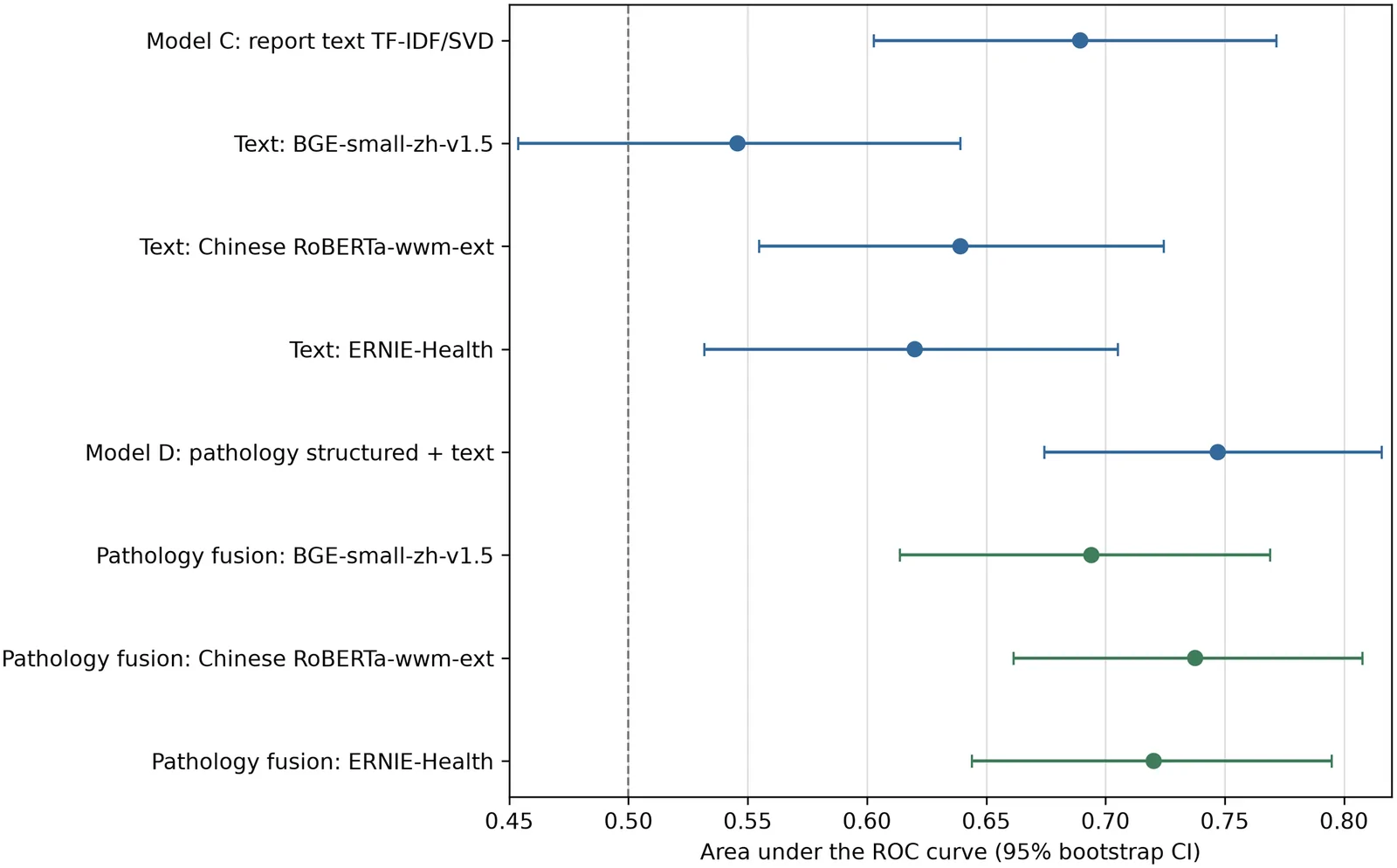
Frozen-encoder NLP baseline comparison. AUC estimates with 95% bootstrap confidence intervals for TF-IDF/SVD, BGE-small-zh-v1.5, Chinese RoBERTa-wwm-ext, and ERNIE-Health text representations, alone and fused with structured pathology.

## Discussion

In this single-center model-development cohort, pretreatment core biopsy pathology reports contained most of the observed predictive signal for pCR. Both rule-extracted pathology phenotypes and patient-level text features discriminated better than the available clinical and preoperative laboratory block. Pathology fusion achieved an internally cross-validated AUC of 0.747, whereas adding clinical-laboratory variables did not improve AUC. These estimates characterize internal performance and should not be interpreted as evidence of transportability or clinical readiness.

The coefficient analysis was consistent with established response biology: lower ER and PR expression and higher HER2 expression and Ki-67 were associated with greater predicted pCR probability. The positive coefficient for the HER2-positive/HR-positive rule-based subtype was also consistent with the observed response distribution. However, coefficient magnitudes in a penalized correlated feature set are not causal effect estimates. The clinical-laboratory AUC below 0.5 should likewise not be interpreted as evidence that inflammatory or chemistry variables are biologically irrelevant. In this modest retrospective dataset, the available fields did not provide stable discrimination, and nested tuning did not reverse that result.

This work differs from prior pathology-report NLP studies conducted in several health systems. Rule-based or machine-learning extraction of breast biomarkers has been reported in the United States, Belgium, South Africa, South Korea, and Canada (22–26), whereas more recent large language model pipelines have been evaluated in French reports and Canadian population-based pathology data (27, 28). Cheligeer and colleagues detected pCR from postoperative reports after neoadjuvant chemotherapy and surgery (28). Their task supports registry and cohort construction; our task instead used only pretreatment biopsy reports as predictors and reserved postoperative information for outcome determination. The geographic breadth of prior extraction studies is encouraging, but their differing languages, templates, and reporting practices also underscore that an institutional rule dictionary cannot be presumed portable.

Our study also complements digital pathology and multimodal prediction studies (6–16). Saednia and colleagues reported strong performance using quantitative pathomic features and machine learning from pretreatment biopsy slides (10) and later proposed a hierarchical self-attention framework using digital histopathology images (11). Digital tumor-infiltrating lymphocyte quantification on pretreatment biopsies has also shown predictive value (12), whereas multimodal imaging-clinical models have reported additional gains when rich imaging data are available (15, 16). Those approaches use pixel-level tissue or MRI information and usually require digitized slides, dedicated imaging workflows, or multi-time-point imaging. In contrast, our report-based approach uses pathology text already produced during routine care. It is therefore lower cost and more accessible for retrospective cohorts, but it cannot recover visual information that was never described in the report. Report-based and image-based methods should be viewed as complementary rather than interchangeable.

The limited gain from multimodal fusion may reflect redundancy with pathology biomarkers, noise in the available clinical-laboratory fields, and inadequate precision to detect a small incremental effect. The 95% CI for the multimodal-versus-pathology-fusion AUC difference (−0.048 to 0.017) includes both modest harm and modest benefit. The laboratory block also reflected preoperative testing rather than a uniform pretreatment time point. Accordingly, this result should be framed as an inconclusive incremental-value analysis rather than proof that clinical or laboratory information has no value.

The TF-IDF/SVD representation performed better than frozen BGE and numerically better than the Chinese biomedical ERNIE-Health encoder, whereas Chinese RoBERTa approached the pathology-fusion performance of TF-IDF/SVD. Chinese pathology reports in this corpus were short and formulaic, and marker names, percentage values, and plus-sign patterns may be captured efficiently by local lexical n-grams. Frozen mean-pooled representations can compress these formatting details, whereas task-specific fine-tuning was not attempted because 64 events were insufficient for defensible end-to-end transformer training. These results do not establish the general superiority of TF-IDF; they show only that the tested frozen encoders did not improve performance under the same low-dimensional, internally validated protocol.

Treatment remains central to interpretation because pCR depends on chemotherapy backbone, anti-HER2 therapy, endocrine exposure, dose intensity, completion, and other treatment factors. The secondary analysis suggested a small numerical benefit from adding standardized regimen components, but the AUC-difference interval included no benefit. Treatment allocation was informed by tumor biology and institutional practice, so the treatment-added model is associational and does not estimate the effect of selecting one regimen over another. Moreover, dose intensity, delays, completion, and consistently defined cycle counts were unavailable, and the immunotherapy and endocrine/CDK4/6 strata were too small for stable treatment-specific inference. The primary estimates should therefore be understood as average predictions across the treatment mix represented in this cohort, not as counterfactual predictions for a particular regimen.

No patient’s treatment should be altered on the basis of this model. Before any clinical application, the extraction pipeline and prediction model would require temporal and independent institutional validation, prospective assessment, calibration updating, and threshold-specific impact analysis. Potential research uses after such validation could include trial enrichment or flagging records for multidisciplinary review, but the present analysis does not identify a patient group for whom standard neoadjuvant treatment should be escalated, de-escalated, or changed.

The study has several additional limitations. First, all estimates arose from repeated cross-validation in one cohort at one institution; repeated resampling does not substitute for external validation, and robustness and transportability remain unknown. Second, the 10-year inclusion period permits temporal drift in report templates, diagnostic terminology, biomarker guidelines, and treatment practice. Institution-specific preanalytical factors, including biopsy sampling, tissue handling, cold ischemia, fixation, processing, antibody clones and assay platforms, scoring thresholds, and pathologist interpretation, may influence both extracted phenotypes and text representations. Third, although the 20-case image audit found a low CER and high critical-marker agreement, it was performed by one internal reviewer and targeted diagnosis/IHC text rather than every header and free-text field. Its precision and scope are therefore limited, and two AR status discrepancies show that mixed-sign expressions and OCR loss of a negative symbol can still affect downstream coding. Fourth, AR remained partly unreported and three HER2 values remained uncertain; documentation-related missingness may not satisfy a missing-at-random assumption, and median imputation cannot recover unrecorded biology. The HER2-resolved sensitivity analysis was therefore included as a targeted coding sensitivity check, not as a complete-biomarker analysis. Fifth, with 64 events and up to 135 transformed features in the treatment-added models, overfitting remains plausible despite penalization, dimension reduction, and fold-contained preprocessing. The nested and unweighted analyses support qualitative stability but remain internal and imprecise. Sixth, treatment was omitted from the primary model, the secondary treatment coding lacked uniform dose and completion information, and the laboratory time point was preoperative rather than under uniform pretreatment. Seventh, pCR was retrospectively curated from routine clinical assessment and study-level record review rather than prospectively adjudicated.

Future work should validate the locked extraction rules against locally annotated reports at another institution and test the complete model in an independent temporal and geographic cohort. A staged framework could then quantify the incremental value of detailed treatment exposure, dose intensity, imaging, digital pathology slides, and molecular data in prespecified blocks. Larger multicenter data would also permit model recalibration, interaction analysis by biological subtype and regimen, and more reliable assessment of clinical thresholds.

## Conclusions

In this single-center development study, pretreatment core biopsy pathology reports carried most of the internally observed predictive signal for pCR. Adding the available clinical and preoperative laboratory variables did not improve discrimination over pathology fusion, whereas treatment variables produced a small, statistically uncertain numerical gain. Frozen pretrained encoders did not outperform the lexical text representation. These findings are hypothesis-generating. Independent extraction and external model validation are required before any clinical interpretation or use.

## Data Availability

The raw data supporting the conclusions of this article will be made available by the authors, without undue reservation.

## Supplementary material

Supplementary File S1 Deidentified report-conversion quality-control summary, extraction-rule dictionary, and variable-coding dictionary.

Supplementary File S2 Rule-extracted structured pathology phenotype table.

Supplementary File S3 TRIPOD+AI working checklist.

Supplementary File S4 Deidentified stratified 40-case manual pathology audit subset and annotated comparison table without source filenames or raw report excerpts.

Supplementary File S5 Model sensitivity-analysis output summary.

Supplementary Table 1 Full baseline and pathology characteristics by pCR status.

Supplementary Table 2 Main model performance and paired bootstrap comparisons.

Supplementary Table 3 Frozen BGE, Chinese RoBERTa, and ERNIE-Health embedding sensitivity analyses.

Supplementary Table 4 HER2-resolved sensitivity analysis.

Supplementary Table 5 Blinded OCR character-accuracy and pathology extraction quality-control audit summary.

Supplementary Table 6 Nested hyperparameter-tuning sensitivity analysis for all five principal models.

Supplementary Table 7 Fold-aggregated structured-pathology coefficient stability and contribution analysis.

Supplementary Table 8 Unweighted-model sensitivity analysis.

Supplementary Table 9 Treatment-regimen coding summary and treatment-added secondary models.
