# Supplementary material for: Prediction of pathological complete response after neoadjuvant therapy in breast cancer using pretreatment core biopsy pathology reports: a pathology-report-driven model with exploratory clinical-laboratory fusion
Source: Front Oncol. 2026 Sep 25;16:1909071. doi: 10.3389/fonc.2026.1909071 (PMC13649482; doi:10.3389/fonc.2026.1909071)
Supplement: Supplementary file 1 [file DataSheet3.docx]

TRIPOD+AI Working Checklist

This working checklist maps the revised manuscript to key TRIPOD+AI reporting domains. It is not a substitute for any journal-provided official form.

| **Domain** | **Status** | **Revised manuscript location** | **Notes** |
| --- | --- | --- | --- |
| Title identifies prediction objective and data source | Complete | Title | Identifies pCR prediction from pretreatment core biopsy reports. |
| Abstract reports design, sample, validation, performance, and limitations | Complete | Abstract | Corrected primary and treatment-added secondary results included. |
| Clinical and research context | Complete | Introduction | Distinguishes risk-stratification research from clinical implementation. |
| Data source and setting | Complete | Methods: Study design and cohort; pathology report conversion | Single-center retrospective cohort, institution, dates, and the revision-stage retrieval of 15 archived reports for 14 existing patients reported; no patients or outcomes changed. |
| Eligibility criteria | Complete | Methods: Eligibility criteria | Inclusion and exclusion criteria and final cohort reported. |
| Outcome definition and adjudication | Complete | Methods: Eligibility criteria | pCR definition and retrospective re-review described. |
| Predictor definition and timing | Complete | Methods: Clinical/laboratory variables; structured extraction | Pretreatment reports and preoperative laboratory timing distinguished. |
| Treatment variables | Complete, post hoc | Methods/Results/Discussion; Supplementary Table S9 | Primary exclusion justified; treatment coding, completeness, secondary models, and limitations reported. |
| Sample size and events | Complete | Methods: Validation and statistical analysis | n=171 and 64 pCR events; no a priori calculation; precision limitations stated. |
| Missing-data handling | Complete | Methods: Structured extraction; model development | Structural negatives separated from true unknowns; ER, PR, and Ki-67 complete after archive retrieval; remaining AR/HER2 uncertainty and training-fold imputation reported. |
| Text conversion and OCR quality | Complete | Methods/Results; Supplementary Table S5 | Processing yield, engine confidence, matched-scope 20-case CER with case-bootstrap CI, and blinded 40-case field agreement reported. |
| Rule-development reproducibility | Complete with limitation | Methods: Structured extraction; Supplementary File S1 | Normalization, patterns, aggregation, and locking are reported; no pathologist formally participated in rule development, and the clinical audit occurred only after locking and did not refine the rules. |
| Predictor selection | Complete | Methods: Model development | No outcome-based univariable selection. |
| Model specification | Complete | Methods: Model development | Elastic Net solver, l1 ratio, C, iterations, and class weighting reported. |
| Hyperparameter tuning | Complete | Methods/Results: Sensitivity analyses | Fixed main parameters and all-model nested tuning reported. |
| Internal validation | Complete | Methods: Validation and statistical analysis | 20 repeats of stratified 5-fold CV and averaged out-of-fold predictions. |
| External validation | Limitation | Discussion | None available; clinical claims restricted accordingly. |
| Performance measures | Complete | Methods/Results; Tables 2-3 | AUC, AP, classification measures, Brier score, bootstrap CIs, and paired differences. |
| Calibration and clinical utility | Complete, exploratory | Methods/Results; Figure 2 | Calibration and decision curves reported without clinical-readiness claims. |
| Model complexity and overfitting | Complete | Methods/Discussion | Dimensions, penalization, reduction, fold-contained processing, and residual risk reported. |
| NLP comparator models | Complete, limited scope | Methods/Results; Figure 7; Supplementary Table S3 | TF-IDF, BGE, Chinese RoBERTa, and ERNIE-Health were compared under one protocol; Chinese MedBERT, BioBERT, and ClinicalBERT were not evaluated. |
| Explainability | Complete for structured pathology, limited for transformed text | Methods/Results; Figure 6; Supplementary Table S7 | Fold-aggregated structured-feature coefficient stability is reported; SVD/PCA components were not assigned semantic biomarker labels. |
| Limitations and transportability | Complete | Discussion | Single center, pre-analytical variation, temporal drift, missingness, OCR, treatment, sample size, and endpoint review. |
| Data availability and privacy | Complete | Data availability statement | Protected source data; deidentified aggregate/derived materials only. |
| Ethics | Complete | Ethics statement | Ethics Committee of Xinxiang Central Hospital; project and approval opinion numbers reported. |
| AI assistance disclosure | Complete | Generative AI statement | Tool role, prohibited uses, author verification, and responsibility stated. |

# Final Verification

Language, citation numbering, numerical consistency, privacy, and rendered layout checks were rerun after insertion of the completed human-audit results.
